# Machine learning based prediction for oncologic outcomes of renal cell carcinoma after surgery using Korean Renal Cell Carcinoma (KORCC) database

**DOI:** 10.1038/s41598-023-30826-2

**Published:** 2023-04-08

**Authors:** Jung Kwon Kim, Sangchul Lee, Sung Kyu Hong, Cheol Kwak, Chang Wook Jeong, Seok Ho Kang, Sung-Hoo Hong, Yong-June Kim, Jinsoo Chung, Eu Chang Hwang, Tae Gyun Kwon, Seok-Soo Byun, Yu Jin Jung, Junghyun Lim, Jiyeon Kim, Hyeju Oh

**Affiliations:** 1 Department of Urology, Seoul National University Bundang Hospital, Seongnam, Korea; 2 Department of Urology, Seoul National University College of Medicine, Seoul, Korea; 3 Department of Urology, Seoul National University Hospital, Seoul, Korea; 4 Department of Urology, Korea University Anam Hospital, Seoul, Korea; 5 Department of Urology, Seoul St. Mary’s Hospital, The Catholic University of Korea, Seoul, Korea; 6 Department of Urology, Chungbuk National University Hospital, Cheongju, Korea; 7 Department of Urology, National Cancer Center, Goyang, Korea; 8 Department of Urology, Chonnam National University Medical School, Gwangju, Korea; 9 Department of Urology, Kyungpook National University Chilgok Hospital, Daegu, Korea; 10 Department of Medical Device Development, Seoul National University College of Medicine, Seoul, Korea; 11 The IMC Lnc., Daegu, Korea

**Keywords:** Medical research, Oncology, Urology

## Abstract

We developed a novel prediction model for recurrence and survival in patients with localized renal cell carcinoma (RCC) after surgery and a novel statistical method of machine learning (ML) to improve accuracy in predicting outcomes using a large Asian nationwide dataset, updated KOrean Renal Cell Carcinoma (KORCC) database that covered data for a total of 10,068 patients who had received surgery for RCC. After data pre-processing, feature selection was performed with an elastic net. Nine variables for recurrence and 13 variables for survival were extracted from 206 variables. Synthetic minority oversampling technique (SMOTE) was used for the training data set to solve the imbalance problem. We applied the most of existing ML algorithms introduced so far to evaluate the performance. We also performed subgroup analysis according to the histologic type. Diagnostic performances of all prediction models achieved high accuracy (range, 0.77–0.94) and F1-score (range, 0.77–0.97) in all tested metrics. In an external validation set, high accuracy and F1-score were well maintained in both recurrence and survival. In subgroup analysis of both clear and non-clear cell type RCC group, we also found a good prediction performance.

## Introduction

The incidence of renal cell carcinoma (RCC) is increasing worldwide. Approximately 76,000 new cases and almost 14,000 deaths from RCC were reported in the US in 2021^1^. In Korea, we also observed the same trend according to the latest cancer incidence statistics from the Korea Central Cancer Registry^2^. Among them, clear cell type RCC represents approximately 70% cases in adults^3^. Estimated 5-year survival rate of localized RCC patients is approximately 90%. However, in about 30% of either recurrence or metastasis cases, the survival rate is drastically reduced^4^. Thus, it is imperative to predict the high-risk group for recurrence in advance and establish a differentiated surveillance protocol for patients who have undergone a curative surgery.

Over the past decades, several nomograms for recurrence and/or survival of localized RCC have been developed and applied in clinical practice^5–8^. Among them, the Kattan nomogram based on pathological T stage, nuclear grade, tumor size, necrosis, vascular invasion, and clinical presentation was the first introduced and widely used model^5,6^. Subsequently, the Leibovich model was developed by Mayo Clinic to estimate the risk of metastasis or recurrence using tumor stage, regional lymph node status, tumor size, nuclear grade and histologic tumor necrosis^7^. The most recently developed model known as the GRANT score was based on patient age, nuclear grade, and pathologic T/N stage^8^. However, these models were developed and validated using a small cohort from a single institution. In addition, they were limited to Western datasets. Moreover, their prediction accuracies were not as high as expected. For most models, their accuracy values were around 0.7^5–8^.

Thus, we tried to develop a novel prediction model for recurrence and survival in patients with localized RCC after surgery using a large Asian nationwide dataset. We also used a novel statistical method of machine learning (ML) to improve accuracy in predicting outcomes.

## Materials and methods

### Ethics statement

The Institutional Review Board (IRB) of Seoul National University Bundang Hospital approved this study (approval number: B-2106-688-108). The requirement for obtaining written informed consent from patients was waived by the IRB due to the retrospective nature of this study. Personal identifiers were completely deleted to ensure that data were analyzed anonymously. Our study was conducted according to the ethical standards of the 1964 Declaration of Helsinki and its later amendments.

### Data sets

The KOrean Renal Cell Carcinoma (KORCC) database was first established in 2011. It had data from eight academic institutions nationwide^9^. Recently, data of each institution were updated from March to June 2021. Subsequently, the updated KORCC database covered data of a total of 10,068 patients who had received surgery for RCC with 206 variables, including demographic, perioperative, pathologic, and survival information.

Model development (n = 4,829) and internal validation (n = 2,070) were performed using data from seven centers except data from Seoul National University Bundang Hospital (SNUBH, n = 3,169). External validation was performed using data from the SNUBH to assess the generality of the model performance. SNUBH was suitable for external validation because of its size and diverse patient population.

All study procedures were performed according to the transparent reporting of a multivariable prediction model for individual prognosis or diagnosis (TRIPOD) recommendations^10^.

All institutions obtained IRB approvals before inputting data into the database. Unified data templates were used for consistent data collection at each institution. Survival data were retrospectively reviewed from medical records or identified from death certificate data.

### Data processing and feature selection

Data pre-processing mainly included processing missing values to obtain a reliable set of data. The missing value imputation process was divided into three aspects: patients, predictors, and statistics. At first, we eliminated patients with missing basic information. Subsequently, we performed predictive analytics for variables including total protein, Hb, creatinine. For this method, we used Euclidean distance to determine the similarity between two values and replace the missing one with similar one. Other missing values were corrected using k-nearest neighbor (KNN)^11^. KNN is non-parametric and instance-based method, and useful for datasets having both qualitative and quantitative attribute values.

**Figure 1:**
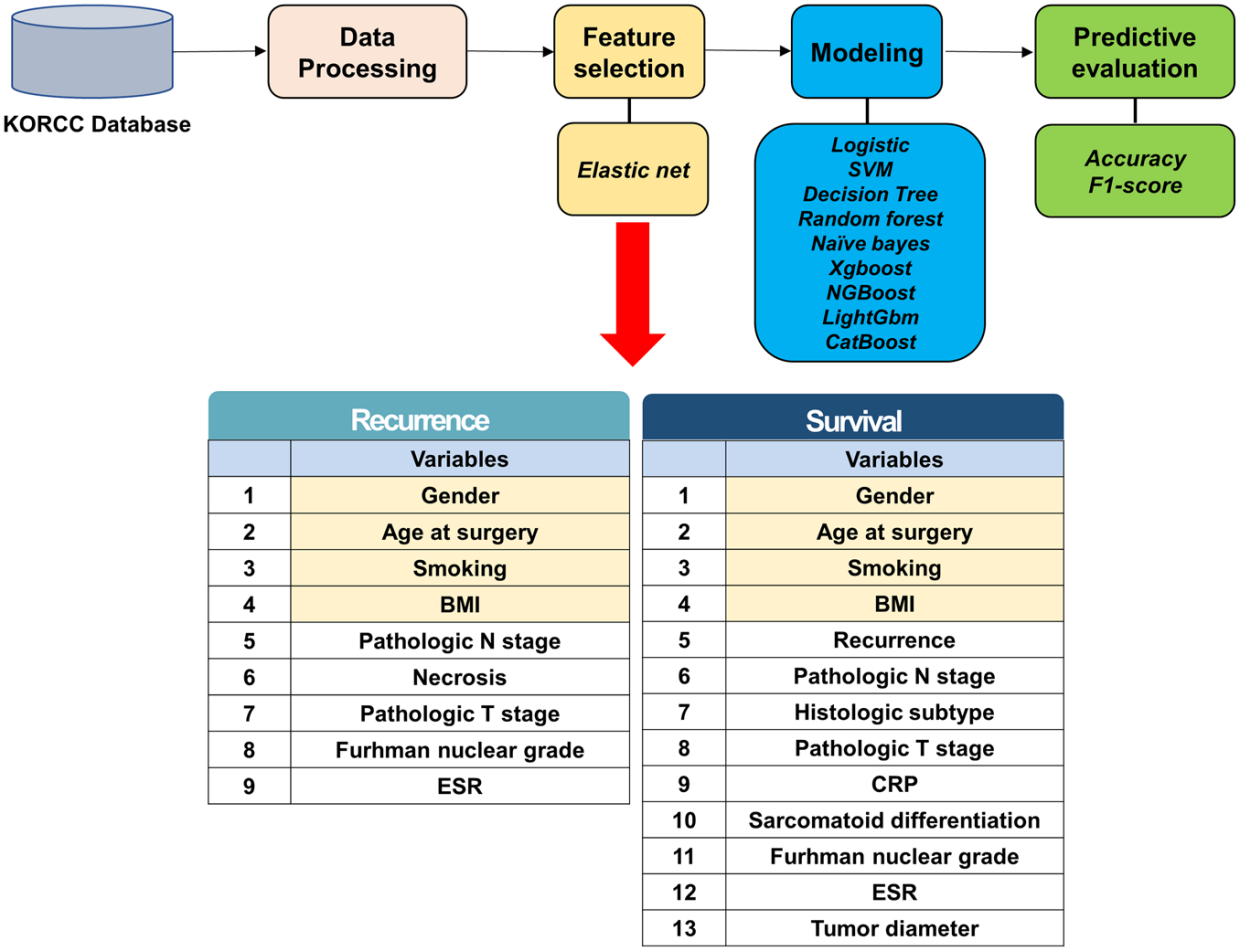
Study flow and final significant variables through feature selection.

After pre-processing, we performed feature selection with an elastic net^12^. Before implementing elastic net model, we defined four default variables that had been considered as the most significant predictors for recurrence and survival: gender, age at surgery, smoking, and BMI^13,14^. Elastic net is known as a hybrid of ridge regression and lasso regularization. Thus, elastic net can generate reduced models by generating zero-valued coefficients. Similar to the lasso, elastic net simultaneously perform automatic variable selection and continuous shrinkage^15^. We subsequently performed a feature importance raking method (Supplemental Fig. 1). Finally, we extracted nine variables for recurrence and 13 variables for survival (Fig. 1).

### Synthetic minority oversampling technique (SMOTE)

Imbalanced data problem is a situation in which data are biased toward one class in applying ML classification algorithms^16^. When modeling using imbalanced data, the ML algorithm attempts to improve the performance by predicting a large number of classes, in which most patients are concentrated, resulting in lower predictability of a small number of classes. Thus, imbalanced data problem should be solved using methods such as oversampling or underdamping. In the current study, we used the SMOTE to the training data set to solve the imbalance problem^17^.

### Statistical analysis and ML model development

We evaluated performances of the following representative ML classification algorithms: logistic regression^18^, kernel support vector machine (SVM)^19^, decision tree^20^, random forest^21^, naïve Bayes (NB)^22^, Extreme Gradient Boosting algorithm (XGBoost)^23^, Natural Gradient Boosting (NGBoost)^24^, LightGbm^25^, and CatBoost^26^. We adopted accuracy and F-1 score to evaluate the prediction performance. The F-1 score is made up of both precision and recall metrics. It is designed to work more accurately on imbalanced data^27^. We also performed subgroup analysis according to histologic type. Non-clear cell type RCC included eight types: papillary, chromophobe, collecting duct, unclassified, multilocular cystic, mixed, Xp11.2 translocation, and clear cell papillary. All statistical analyses were performed using commercially available software (IBM SPSS Statistics ver. 21.0 and Python ver. 3.7.6).

### Ethics statement

The Institutional Review Board (IRB) of Seoul National University Bundang Hospital approved this study (approval number: B-2106–688-108).

### Informed consent to patients

The waiver of the informed consent requirement was approved by the local ethics committee of Seoul National University Bundang Hospital considering the retrospective study design involving anonymized data.

## Results

### Patient characteristics

Distribution of data sets before and after SMOTE for recurrence (n = 6,717) and survival (n = 5,730) is described in Table 1. The ratio of training set to test set was 7:3. Overall survival rates at 3, 5, and 10 years were 94.2%, 90.6%, and 71.9%, respectively; and the recurrence-free rates were 85.2%, 78.8% and 45.3%, respectively.

**Table 1:** Distribution of data sets before and after synthetic minority oversampling technique application (SMOTE).

			Training set (70%)		Test set (30%)	
Recurrence (n = 6717)			No	Yes	No	Yes
Total group	3-year	Before (Raw data)	3031	521	1298	233
		After (SMOTE)	10,420	2605		
	5-year	Before (Raw data)	2281	621	987	256
		After (SMOTE)	12,420	3105		
	10-year	Before (Raw data)	589	688	236	310
		After (SMOTE)	1178	1178		

			Training set (70%)		Test set (30%)	
Survival (n = 5730)			Alive	Death	Alive	Death
Total group	3-year	Before (Raw data)	2871	173	1226	78
		After (SMOTE)	3460	865		
	5-year	Before (Raw data)	2232	207	924	120
		After (SMOTE)	4140	1035		
	10-year	Before (Raw data)	621	229	273	120
		After (SMOTE)	598	598		

Subsequently, we compared patient characteristics and distribution of each variable for recurrence and survival (Table 2). In a comparative analysis between recurrence and non-recurrence groups, we found several significantly different variables except for four default variables (gender, age at surgery, smoking, and BMI): Eastern Cooperative Oncology Group (ECOG) performance status, symptoms at diagnosis, transfusion, pathologic T/N stages, sarcomatoid differentiation, necrosis, lymphovascular invasion (LVI), and Fuhrman nuclear grade (all *p* < 0.05). In terms of survival, ECOG performance status, symptoms at diagnosis, transfusion, pathologic T/N stages, sarcomatoid differentiation, necrosis, LVI, histologic type, Fuhrman nuclear grade, and recurrence were significant variables (all *p* < 0.05).

**Table 2:** Baseline characteristics.

	Recurrence		Survival	
Variable	No (n = 5698)	Yes (n = 1019)	Alive (n = 5279)	Death (n = 451)
Age, years, mean (SD)	55.5 (12.7)	58.3 (11.6)	54.8 (12.4)	59.5 (11.7)
Gender, male, n (%)	4024 (70.6)	754 (74.0)	3710 (70.3)	340 (75.4)
BMI, kg/m^2^, mean (SD)	24.8 (3.3)	23.9 (3.1)	24.9 (3.3)	23.5 (3.1)
DM, n (%)	878 (15.4)	205 (20.1)	783 (14.8)	97 (21.5)
HTN, n (%)	2336 (41.0)	463 (45.4)	2162 (41.0)	204 (45.2)
CKD, n (%)	142 (2.5)	25 (2.5)	129 (2.4)	8 (1.8)
Smoking status, n (%)				
Non-smoker	3552 (62.3)	682 (66.9)	3304 (62.6)	333 (73.8)
Ex-smoker	1199 (21.0)	154 (15.1)	1120 (21.2)	48 (10.6)
Current smoker	947 (16.6)	183 (18.0)	855 (16.2)	70 (15.5)
ECOG, n (%)				
0	4214 (74.0)	561 (55.1)	3980 (75.4)	169 (37.5)
1	1012 (17.8)	262 (25.7)	892 (16.9)	123 (27.3)
≥ 2	472 (8.2)	196 (19.2)	407 (7.7)	159 (35.3)
Symptoms at diagnosis, n (%)	1044 (18.3)	448 (44.0)	954 (18.1)	220 (48.8)
Surgical modality, n (%)				
Open	1837 (32.2)	608 (59.7)	1594 (30.2)	339 (75.2)
Laparoscopic	1902 (33.4)	288 (28.2)	1654 (31.4)	98 (21.7)
Robotic	1959 (34.4)	123 (12.1)	2026 (38.4)	13 (2.9)
Transfusion, n (%)				
Intra-operative	223 (3.9)	193 (18.9)	196 (3.7)	83 (18.4)
Post-operative	199 (3.5)	113 (11.1)	202 (3.8)	56 (12.4)
Perioperative complications, n (%)	372 (6.5)	119 (11.7)	367 (7.0)	72 (16.0)
Pathologic T stage, n (%)				
1a	3701 (65.0)	181 (17.8)	3347 (63.4)	53 (11.8)
1b	1186 (20.8)	201 (19.7)	1095 (20.7)	91 (20.2)
2	327 (5.7)	177 (17.4)	335 (6.3)	77 (17.1)
≥ 3	484 (8.5)	460 (45.1)	502 (9.5)	230 (51.0)
Pathologic N stage, n (%)				
N0/Nx	5651 (99.2)	904 (88.4)	5236 (99.2)	366 (81.1)
N1	47 (0.8)	118 (11.6)	43 (0.8)	85 (18.9)
Positive surgical margin, n (%)	33 (0.6)	9 (0.9)	34 (0.6)	5 (1.1)
Sarcomatoid differentiation, n (%)	87 (1.5)	116 (11.4)	126 (2.4)	50 (11.1)
Necrosis, n (%)	519 (9.1)	306 (40.0)	491 (9.2)	126 (27.9)
LVI, n (%)	158 (2.8)	181 (17.8)	180 (3.4)	69 (15.3)
Histologic type, n (%)				
Clear cell	4740 (83.2)	860 (84.4)	4404 (83.4)	355 (78.7)
Non-clear cell				
Papillary	439 (7.7)	72 (7.1)	389 (7.4)	40 (8.9)
Chromophobe	385 (6.8)	25 (2.5)	358 (6.8)	13 (2.9)
Collecting duct	9 (0.2)	17 (1.7)	11 (0.2)	11 (2.4)
Unclassified	40 (0.7)	25 (2.5)	39 (0.7)	20 (4.4)
Multilocular cystic RCC	26 (0.5)	1 (0.1)	24 (0.5)	0 (0.0)
Mixed	19 (0.3)	7 (0.7)	14 (0.3)	3 (0.7)
Xp11.2 translocation RCC	13 (0.2)	10 (1.0)	15 (0.3)	7 (1.6)
Clear cell papillary RCC	27 (0.5)	2 (0.2)	25 (0.5)	2 (0.4)
Fuhrman nuclear grade, n (%)				
1/2	3026 (53.1)	270 (26.5)	2677 (50.7)	95 (21.1)
3/4	2672 (46.9)	749 (73.5)	2602 (49.3)	356 (78.9)
Recurrence, n (%)	–	–	408 (7.7)	79 (17.5)

### Prediction model performance and external validation

**Figure 2:**
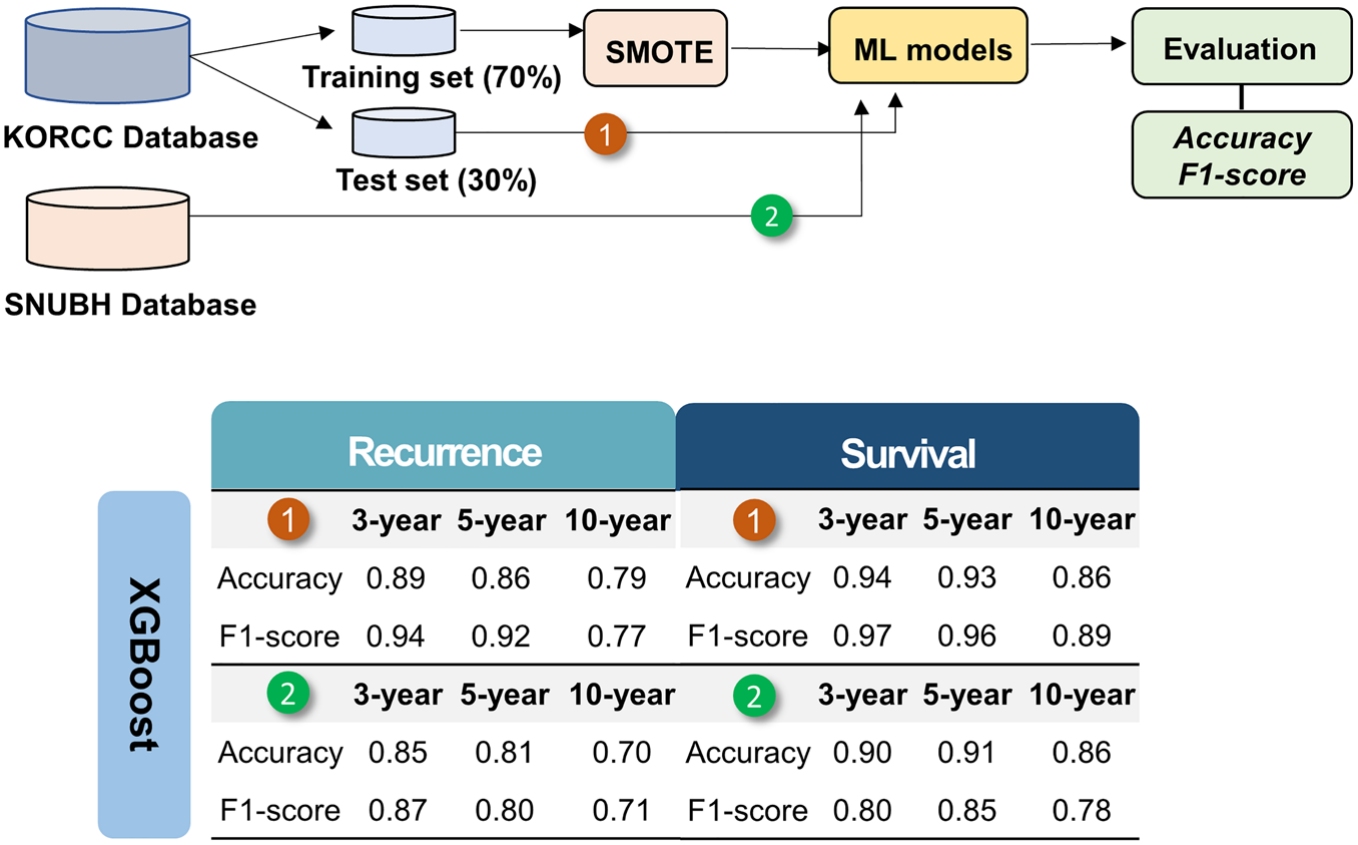
Compositions of database and results of (1) internal and (2) external validation for recurrence and survival.

Diagnostic performance of several machine learning algorithms for the prediction of 3-, 5-, and 10-year recurrence and survival are listed in Table 3. All models achieved very high accuracy (range, 0.77–0.94) and F1-score (range, 0.77–0.97) in all tested metrics. Subsequently, external validation with a SNUBH dataset (n = 3,169) was performed using all models (Fig. 2). High accuracy and F1-score were well maintained in external validation in both recurrence and survival (Supplemental Table 1).

**Table 3:** Diagnostic performance of machine learning algorithms for the prediction of recurrence and survival.

		Recurrence			Survival		
Model	Method	3-year	5-year	10-year	3-year	5-year	10-year
Logistic Regression	Accuracy	0.90	0.87	0.81	0.93	0.94	0.87
	F1-score	0.94	0.92	0.80	0.96	0.96	0.90
SVM	Accuracy	0.90	0.86	0.78	0.94	0.93	0.86
	F1-score	0.94	0.92	0.77	0.97	0.96	0.90
Decision Tree	Accuracy	0.86	0.85	0.77	0.90	0.90	0.78
	F1-score	0.91	0.90	0.78	0.95	0.95	0.82
Random Forest	Accuracy	0.88	0.84	0.78	0.93	0.92	0.84
	F1-score	0.93	0.89	0.77	0.96	0.95	0.88
Naïve bayes	Accuracy	0.88	0.87	0.77	0.91	0.91	0.88
	F1-score	0.93	0.92	0.77	0.95	0.95	0.91
XGBoost	Accuracy	0.89	0.86	0.79	0.94	0.93	0.86
	F1-score	0.94	0.92	0.77	0.97	0.96	0.89
NGBoost	Accuracy	0.88	0.86	0.80	0.93	0.92	0.86
	F1-score	0.93	0.92	0.80	0.96	0.95	0.89
LightGbm	Accuracy	0.88	0.84	0.78	0.90	0.91	0.84
	F1-score	0.93	0.90	0.78	0.94	0.95	0.85
CatBoost	Accuracy	0.89	0.87	0.79	0.94	0.93	0.86
	F1-score	0.94	0.92	0.78	0.97	0.96	0.89

### Subgroup analysis

In subgroup analysis according to the histologic type (clear vs. non-clear cell type RCC), dataset distribution before and after SMOTE for recurrence and survival is described in Supplemental Table 2. Consequently, we also found very high accuracy (range, 0.64–0.91) and F1-score (range, 0.72–0.94) in all tested metrics (Supplemental Tables 3 and 4).

## Discussion

Using the original KORCC database^9^, two recent studies have been reported^28,29^. At first, Byun et al.^28^ assessed the prognosis of non-metastatic clear cell RCC using a deep learning-based survival predictions model. Harrel’s C-indices of DeepSurv for recurrence and cancer-specific survival were 0.802 and 0.834, respectively. More recently, Kim et al.^29^ developed ML-based algorithm predicting the probability of recurrence at 5 and 10 years after surgery. The highest area under the receiver operating characteristic curve (AUROC) was obtained from the naïve Bayes (NB) model, with values of 0.836 and 0.784 at 5 and 10 years, respectively.

In the current study, we used the updated KORCC database. It now contains clinical data of more than 10,000 patients. To the best of our knowledge, this is the largest dataset in Asian population with RCC. With this dataset, we could develop much more accurate models with very high accuracy (range, 0.77–0.94) and F1-score (range, 0.77–0.97, Table 3). The accuracy values were relatively high compared to the previous models, including the Kattan nomogram, Leibovich model, the GRANT score, which were around 0.7^5–8^. Among them, the Kattan nomogram was developed using a cohort of 601 patients with clinically localized RCC, and the overall C-index was 74%^5^. In a subsequent analysis with the same patient group using an additional prognostic variables including tumor necrosis, vascular invasion, and tumor grade, the C-index was as high as 82%^30^. Their prediction accuracies were not as high as ours yet.

In addition, we could include short-term (3-year) recurrence and survival data, which would be helpful for developing more sophisticated surveillance strategy. The other strength of current study was that most algorithms introduced so far had been applied^18–26^, showing relatively consistent performance with high accuracy. Finally, we also performed an external validation by using a separate (SNUBH) cohort, and achieved well maintained high accuracy and F1-score in both recurrence and survival (Fig. 2). External validation of prediction models is essential, especially in case of using the multi-institutional dataset, to ensure and correct for differences between institutions.

AUROC has been mostly used as the standard evaluating performance of prediction models^5–8,29^. However, AUROC weighs changes in sensitivity and specificity equally without considering clinically meaningful information^6^. In addition, the lack of ability to compare performance of different ML models is another limitation of AUROC technique^31^. Thus, we adopted accuracy and F1-score instead of AUROC as evaluation metrics. F1-score, in addition to SMOTE^17^, is used as better accuracy metrics to solve the imbalanced data problems^27^.

RCC is not a single disease, but multiple histologically defined cancers with different genetic characteristics, clinical courses, and therapeutic responses^32^. With regard to metastatic RCC, the International Metastatic Renal Cell Carcinoma Database Consortium and the Memorial Sloan Kettering Cancer Center risk model have been extensively validated and widely used to predict survival outcomes of patients receiving systemic therapy^33,34^. However, both risk models had been developed without considering histologic subtypes. Thus, the predictive performance was presumed to have been strongly affected by clear cell type (predominant histologic subtype) RCC. Interestingly, in our previous study using the Korean metastatic RCC registry, we found the both risk models reliably predicted progression and survival even in non-clear cell type RCC^35^. In the current study, after performing subgroup analysis according to the histologic type (clear vs. non-clear cell type RCC), we also found very high accuracy and F1-score in all tested metrics (Supplemental Tables 3 and 4). Taking together, these findings suggest that the prognostic difference between clear and non-clear cell type RCC seems to be offset both in metastatic and non-metastatic RCC. Further effort is needed to develop and validate a sophisticated prediction model for individual subtypes of non-clear cell type RCC.

The current study had several limitations. First, due to the paucity of long-term follow-up cases at 10 years, data imbalance problem could not be avoided. Subsequently, recurrence-free rate at 10-year was reported only to be 45.3%. In the majority of patients, further long-term follow up had not been performed in case of no evidence of disease at five years. However, we adopted both SMOTE and F1-score to solve these imbalanced data problems. The retrospective design of this study was also an inherent limitation. Another limitation was that the developed prediction model only included the Korean population. Validation of the model using data from other countries and races is also needed. In regard of non-clear cell type RCC, the current study cohort is still relatively small due to the rarity of the disease, we could not avoid integrating each subtype and analyzing together. Thus, further studies is still needed to develop and validate a prediction model for each subtypes. In addition, the lack of more accurate classifiers such as cross-validation and bootstrapping is another limitation of current study. Finally, the web-embedded deployment of model should be followed to improve accessibility and transportability.

## Conclusions

A novel ML algorithm for predicting recurrence and survival in localized RCC patients after surgery was successfully developed and validated using the updated KORCC database. This prediction model is anticipated to offer a differentiated surveillance protocol. It will be a useful tool for patient counseling.

## Supplementary Information

Supplementary Information 1.

Supplementary Information 2.

## Data Availability

All data enquiries can be directed to the corresponding author.
