# Supplementary figures and images for: Machine learning based prediction for oncologic outcomes of renal cell carcinoma after surgery using Korean Renal Cell Carcinoma (KORCC) database
Source: Sci Rep. 2023 Apr 8;13:5778. doi: 10.1038/s41598-023-30826-2 (PMC10082844; doi:10.1038/s41598-023-30826-2)

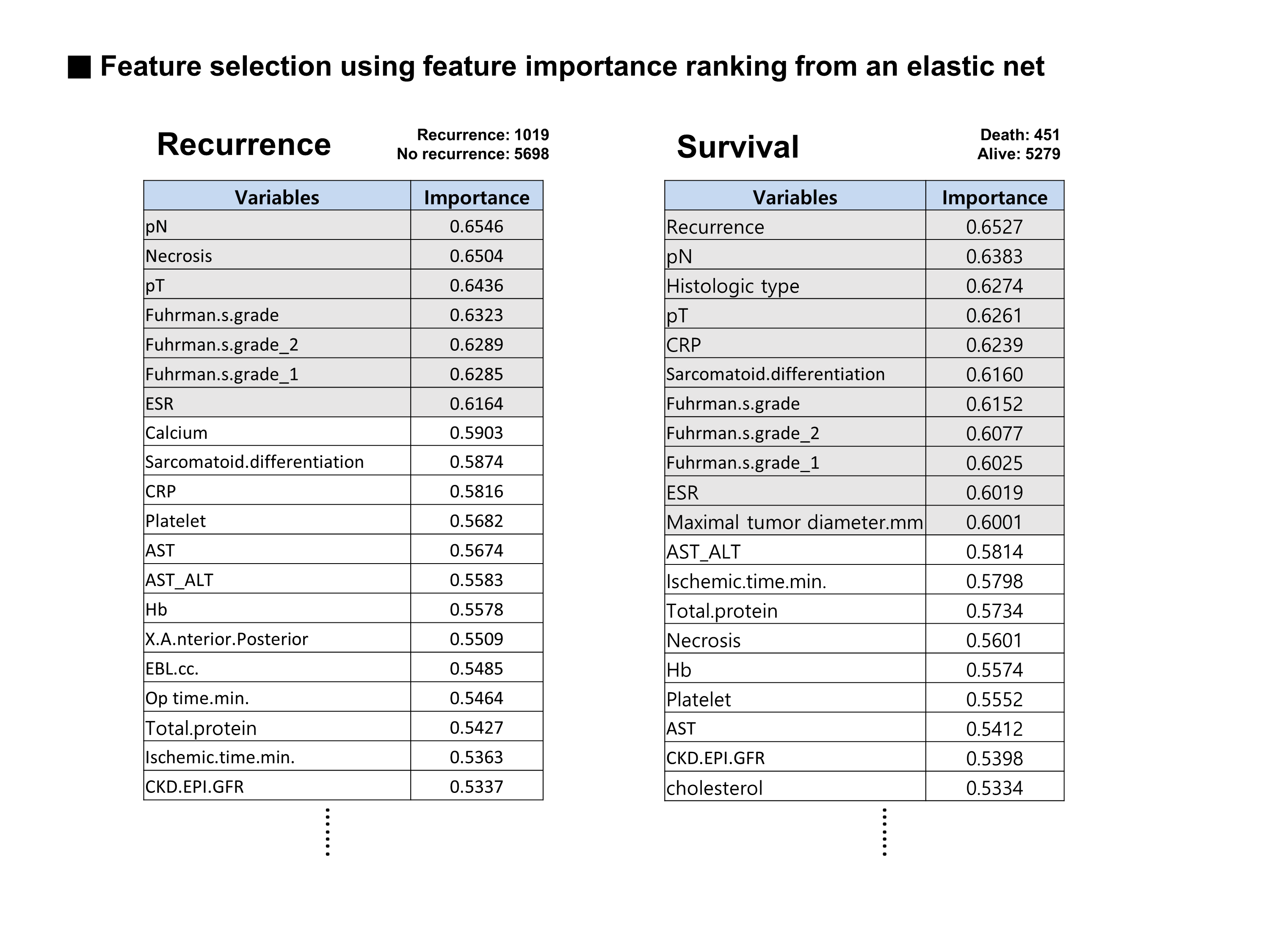

Supplement: Supplementary file 1 — Supplementary Information 1. [file 41598_2023_30826_MOESM1_ESM.tif]
