# Supplementary material for: Machine learning based prediction for oncologic outcomes of renal cell carcinoma after surgery using Korean Renal Cell Carcinoma (KORCC) database
Source: Sci Rep. 2023 Apr 8;13:5778. doi: 10.1038/s41598-023-30826-2 (PMC10082844; doi:10.1038/s41598-023-30826-2)
Supplement: Supplementary file 2 — Supplementary Information 2. [file 41598_2023_30826_MOESM2_ESM.docx]

Supplemental Table 1. Diagnostic performance of machine learning algorithms in the independent external validation cohort (SNUBH dataset, n=3169)

|  |  | **Recurrence** | | | **Survival** | | |
| --- | --- | --- | --- | --- | --- | --- | --- |
| **Model** | **Method** | **3-year** | **5-year** | **10-year** | **3-year** | **5-year** | **10-year** |
| **Logistic Regression** | Accuracy | 0.83 | 0.82 | 0.77 | 0.89 | 0.89 | 0.82 |
|  | F1-score | 0.94 | 0.85 | 0.70 | 0.87 | 0.86 | 0.79 |
| **SVM** | Accuracy | 0.84 | 0.80 | 0.71 | 0.88 | 0.85 | 0.79 |
|  | F1-score | 0.94 | 0.79 | 0.70 | 0.80 | 0.79 | 0.77 |
| **Decision Tree** | Accuracy | 0.85 | 0.83 | 0.75 | 0.87 | 0.85 | 0.75 |
|  | F1-score | 0.90 | 0.80 | 0.70 | 0.81 | 0.79 | 0.77 |
| **Random Forest** | Accuracy | 0.83 | 0.79 | 0.70 | 0.87 | 0.85 | 0.80 |
|  | F1-score | 0.92 | 0.88 | 0.75 | 0.90 | 0.85 | 0.79 |
| **Naïve bayes** | Accuracy | 0.83 | 0.80 | 0.77 | 0.88 | 0.85 | 0.82 |
|  | F1-score | 0.93 | 0.85 | 0.73 | 0.82 | 0.80 | 0.77 |
| **XGBoost** | Accuracy | 0.85 | 0.81 | 0.70 | 0.92 | 0.91 | 0.86 |
|  | F1-score | 0.97 | 0.80 | 0.71 | 0.90 | 0.85 | 0.78 |
| **NGBoost** | Accuracy | 0.86 | 0.79 | 0.72 | 0.89 | 0.88 | 0.86 |
|  | F1-score | 0.91 | 0.85 | 0.81 | 0.85 | 0.84 | 0.81 |
| **LightGbm** | Accuracy | 0.88 | 0.83 | 0.78 | 0.85 | 0.82 | 0.75 |
|  | F1-score | 0.92 | 0.81 | 0.75 | 0.82 | 0.81 | 0.80 |
| **CatBoost** | Accuracy | 0.85 | 0.82 | 0.79 | 0.89 | 0.85 | 0.82 |
|  | F1-score | 0.90 | 0.85 | 0.78 | 0.89 | 0.81 | 0.79 |

Supplemental Table 2. Distribution of data sets before and after synthetic minority oversampling technique application (SMOTE) – subgroup of clear cell and non-clear cell renal cell carcinoma group.

|  | | | **Training set (70%)** | | **Test set (30%)** | |
| --- | --- | --- | --- | --- | --- | --- |
| **Recurrence** | | | **No** | **Yes** | **No** | **Yes** |
| **Clear cell type RCC**  **(n=5629)** | 3-year | Before (Raw data) | 2558 | 435 | 1097 | 185 |
|  |  | After (SMOTE) | 8700 | 2175 |  |  |
|  | 5-year | Before (Raw data) | 1097 | 535 | 849 | 197 |
|  |  | After (SMOTE) | 10700 | 2675 |  |  |
|  | 10-year | Before (Raw data) | 506 | 583 | 208 | 258 |
|  |  | After (SMOTE) | 1012 | 1012 |  |  |
| **Non-clear cell type RCC (n=1088)** | 3-year | Before (Raw data) | 468 | 91 | 206 | 33 |
|  |  | After (SMOTE) | 1820 | 455 |  |  |
|  | 5-year | Before (Raw data) | 356 | 104 | 156 | 41 |
|  |  | After (SMOTE) | 2080 | 520 |  |  |
|  | 10-year | Before (Raw data) | 72 | 116 | 39 | 41 |
|  |  | After (SMOTE) | 144 | 144 |  |  |
| **Survival** | | | **Alive** | **Death** | **Alive** | **Death** |
| **Clear cell type RCC**  **(n=4786)** | 3-year | Before (Raw data) | 2425 | 131 | 1031 | 64 |
|  |  | After (SMOTE) | 2620 | 655 |  |  |
|  | 5-year | Before (Raw data) | 1864 | 182 | 798 | 78 |
|  |  | After (SMOTE) | 3640 | 910 |  |  |
|  | 10-year | Before (Raw data) | 549 | 232 | 234 | 100 |
|  |  | After (SMOTE) | 464 | 464 |  |  |
| **Non-clear cell type RCC (n=944)** | 3-year | Before (Raw data) | 451 | 39 | 190 | 17 |
|  |  | After (SMOTE) | 780 | 195 |  |  |
|  | 5-year | Before (Raw data) | 347 | 47 | 147 | 20 |
|  |  | After (SMOTE) | 940 | 235 |  |  |
|  | 10-year | Before (Raw data) | 82 | 58 | 29 | 29 |
|  |  | After (SMOTE) | 116 | 116 |  |  |

Supplemental Table 3. Diagnostic performance of machine learning algorithms for the prediction of recurrence - subgroup of clear cell and non-clear cell renal cell carcinoma group.

|  |  | **Clear cell type** | | | **Non-clear cell type** | | |
| --- | --- | --- | --- | --- | --- | --- | --- |
| **Model** | **Method** | **3-year** | **5-year** | **10-year** | **3-year** | **5-year** | **10-year** |
| **Logistic Regression** | Accuracy | 0.90 | 0.86 | 0.67 | 0.90 | 0.87 | 0.64 |
|  | F1-score | 0.94 | 0.92 | 0.73 | 0.94 | 0.92 | 0.72 |
| **SVM** | Accuracy | 0.89 | 0.85 | 0.80 | 0.89 | 0.87 | 0.81 |
|  | F1-score | 0.94 | 0.91 | 0.79 | 0.94 | 0.92 | 0.82 |
| **Decision Tree** | Accuracy | 0.87 | 0.87 | 0.77 | 0.87 | 0.86 | 0.75 |
|  | F1-score | 0.92 | 0.92 | 0.77 | 0.92 | 0.92 | 0.75 |
| **Random Forest** | Accuracy | 0.87 | 0.85 | 0.81 | 0.88 | 0.84 | 0.85 |
|  | F1-score | 0.92 | 0.90 | 0.80 | 0.93 | 0.89 | 0.86 |
| **Naïve bayes** | Accuracy | 0.88 | 0.86 | 0.79 | 0.90 | 0.89 | 0.84 |
|  | F1-score | 0.93 | 0.92 | 0.80 | 0.94 | 0.93 | 0.85 |
| **XGBoost** | Accuracy | 0.89 | 0.85 | 0.79 | 0.91 | 0.87 | 0.79 |
|  | F1-score | 0.94 | 0.91 | 0.79 | 0.95 | 0.92 | 0.80 |
| **NGBoost** | Accuracy | 0.89 | 0.87 | 0.81 | 0.89 | 0.87 | 0.80 |
|  | F1-score | 0.94 | 0.92 | 0.81 | 0.94 | 0.92 | 0.80 |
| **LightGbm** | Accuracy | 0.87 | 0.87 | 0.77 | 0.87 | 0.85 | 0.78 |
|  | F1-score | 0.93 | 0.92 | 0.76 | 0.92 | 0.91 | 0.76 |
| **CatBoost** | Accuracy | 0.89 | 0.86 | 0.82 | 0.89 | 0.88 | 0.86 |
|  | F1-score | 0.94 | 0.92 | 0.81 | 0.94 | 0.93 | 0.86 |

Supplemental Table 4. Diagnostic performance of machine learning algorithms for the prediction of survival - subgroup of clear cell and non-clear cell renal cell carcinoma group.

|  |  | **Clear cell type** | | | **Non-clear cell type** | | |
| --- | --- | --- | --- | --- | --- | --- | --- |
| **Model** | **Method** | **3-year** | **5-year** | **10-year** | **3-year** | **5-year** | **10-year** |
| **Logistic Regression** | Accuracy | 0.94 | 0.94 | 0.87 | 0.94 | 0.92 | 0.84 |
|  | F1-score | 0.97 | 0.97 | 0.91 | 0.97 | 0.96 | 0.84 |
| **SVM** | Accuracy | 0.94 | 0.94 | 0.86 | 0.94 | 0.91 | 0.88 |
|  | F1-score | 0.97 | 0.96 | 0.89 | 0.97 | 0.95 | 0.88 |
| **Decision Tree** | Accuracy | 0.94 | 0.91 | 0.87 | 0.93 | 0.87 | 0.88 |
|  | F1-score | 0.97 | 0.95 | 0.90 | 0.96 | 0.93 | 0.88 |
| **Random Forest** | Accuracy | 0.94 | 0.92 | 0.87 | 0.93 | 0.88 | 0.84 |
|  | F1-score | 0.97 | 0.95 | 0.90 | 0.96 | 0.93 | 0.83 |
| **Naïve bayes** | Accuracy | 0.92 | 0.92 | 0.89 | 0.92 | 0.90 | 0.86 |
|  | F1-score | 0.96 | 0.96 | 0.92 | 0.95 | 0.94 | 0.86 |
| **XGBoost** | Accuracy | 0.94 | 0.94 | 0.86 | 0.95 | 0.92 | 0.86 |
|  | F1-score | 0.97 | 0.97 | 0.89 | 0.97 | 0.95 | 0.85 |
| **NGBoost** | Accuracy | 0.94 | 0.93 | 0.89 | 0.92 | 0.89 | 0.86 |
|  | F1-score | 0.97 | 0.96 | 0.92 | 0.95 | 0.94 | 0.86 |
| **LightGbm** | Accuracy | 0.93 | 0.92 | 0.85 | 0.93 | 0.90 | 0.91 |
|  | F1-score | 0.96 | 0.96 | 0.89 | 0.96 | 0.94 | 0.91 |
| **CatBoost** | Accuracy | 0.94 | 0.93 | 0.88 | 0.93 | 0.90 | 0.88 |
|  | F1-score | 0.97 | 0.96 | 0.91 | 0.96 | 0.94 | 0.88 |
